# Infant feeding decisions among people living with human immunodeficiency virus in the United States: a pilot study

**DOI:** 10.3389/frph.2026.1804342

**Published:** 2026-06-18

**Authors:** Laura Cox, Sarah A. Gutin, Ifeyinwa V. Asiodu

**Affiliations:** 1 Department of Family Health Care Nursing, School of Nursing, University of California, San Francisco, CA, United States; 2 Department of Community Health Systems, School of Nursing, University of California, San Francisco, CA, United States; 3 Division of Prevention Sciences, Dept. of Medicine, University of California, San Francisco, CA, United States

**Keywords:** breastfeeding, formula feeding, HIV, infant feeding, postpartum, pregnancy

## Abstract

**Introduction:**

The benefits of breastfeeding for the lactating person and infant have been well established; however, for those living with HIV, the option to provide human milk has been contraindicated. In January 2023, the US national guidance was revised so breastfeeding can be supported through a shared decision-making process for virally suppressed individuals living with HIV. Few research studies have explored maternal experiences related to infant feeding decisions among people living with HIV (PWH) in the US.

**Methods:**

A qualitative, modified critical ethnographic method was used to conduct in-depth interviews with pregnant, postpartum, or parenting persons currently living with HIV in the US. Semi-structured interviews were completed between October 2023 and March 2024. Analyses consisted of a grounded theory technique and thematic analysis.

**Results:**

A total of ten pregnant (*n* = 3), postpartum (*n* = 2), or parenting (*n* = 5) (aged 27–40 years) participants took part. Participants reported living with HIV between 4 and 28 years, with all participants currently taking anti-retroviral medication. Four central themes emerged: the need for support, fear of HIV transmission, the impact of HIV-related stigma, and the desire for feeding options. All participants emphasized the importance of support from family and friends, healthcare providers, and the community. Those who chose formula expressed fear of HIV transmission as the primary reason for not breastfeeding. Fear of transmission was an important motivating factor for infant medication prophylaxis for those who breastfed. Participants described experiences of internalized, anticipated, and experienced stigma from providers, family, and community members when discussing infant feeding decisions and disclosing their status. Finally, all participants wanted infant feeding options to be discussed with healthcare providers, regardless of their infant feeding intention.

**Discussion:**

Multiple factors influence the infant feeding decisions of PWH in the US. Due to these factors, there is a need for an informed, free-choice model that prioritizes unbiased information and situates feeding decisions in the hands of PWH. Further research is needed to ensure PWH are fully supported in their infant feeding decisions.

## 1 Introduction

The benefits of human milk for both lactating persons and infants have been well established (1–4). Recent studies have found that the initiation of lactation is associated with lowering the odds of infant mortality and the severity of a myriad of diseases in infants (3, 5–8), which include asthma, type 1 diabetes, childhood inflammatory bowel disease, heart disease and sleep-disordered breathing (1, 9). For the lactating person, the benefits of lactation begin soon after birth and continue throughout their life course. Following childbirth, breastfeeding helps to reduce the risk of postpartum hemorrhage, anemia, and depression when breastfeeding goes well (10, 11). Beyond postpartum, breastfeeding provides a reduced risk of type 2 diabetes, hypertension, hyperlipidemia, cardiovascular disease, osteoporosis, premenopausal breast cancer, and ovarian cancer (11, 12).

Despite the many benefits of breastfeeding for both women and infants, breastfeeding has been contraindicated for those living with Human Immunodeficiency Virus (HIV) in the United States (US) since 1985. Since the 1990s, however, research has been ongoing to understand the risk of HIV transmission via birth and breastfeeding, with the majority of this research generated from resource-poor countries where access to antiretroviral therapy (ART) was more limited (13–16). Research has established that exclusive lactation in conjunction with continued ART decreases the rate of HIV transmission to less than 1% (17, 18), while also reducing the risk of infant morbidity and mortality. As a result, in 2009, the World Health Organization (WHO) released a position statement recommending six months of exclusive breastfeeding and up to 12 months of breastfeeding with complementary foods for those who have continued access to ART (19). However, despite advances in ART and evidence of low perinatal transmission rates, for the past 38 years, lactating people living with HIV (PWH) in the US have faced restrictions such as mandated alternate infant feeding methods and a lack of birth autonomy (20–25). If infant feeding guidance was not adhered to, lactating PWH often endured criminalization and the involvement of child protective services (CPS) (26–29).

While the landscape of HIV infections varies globally, in the US, infection rates continue to disproportionately impact women of color who have the highest HIV prevalence rates among women (30–32). Factors such as systemic racism, poverty, unstable housing, poor educational attainment, and mass incarceration continue to exacerbate disparities in access to ART medication and culturally responsive care, perpetuate stigma, and erode trust in the healthcare system, generating inequitable access to screening and treatment among Black, Indigenous, and other racially minoritized communities (33–39). Moreover, targeted marketing by formula companies and the historical impact of slavery (40, 41) contribute to the lower exclusive breastfeeding rates at 6 months among Black women in the US (24.2% in 2022), well below the Healthy People 2030 goal of 42.4% (42–45). As a result, the restrictions on bodily autonomy and HIV related stigma have influenced infant feeding decisions and infringed upon the reproductive rights of PWH (25, 46, 47).

Recently, there has been a nationwide paradigm shift regarding breastfeeding and HIV. In January 2023, the Department of Health and Human Services (DHHS) Panel on Treatment of HIV During Pregnancy and Prevention of Perinatal Transmission updated its guidance to recommend a patient and provider shared decision-making model for infant feeding that includes evidence-based information on the risk of transmission and the benefit of breastfeeding while living with HIV (48). The guidelines stipulate that individuals who have an undetectable viral load in pregnancy and continue to take ART should be counseled on breastfeeding or replacement feeding and receive support in their decision. While several studies from the international community have aimed to understand infant feeding decisions while living with HIV, due to contraindications to human milk feeding in the US, there is little qualitative US literature regarding the experience of those making infant feeding decisions while living with HIV (49–52). To fill this gap, and in light of the recent policy shift in infant feeding guidelines in the US, we conducted a study with pregnant, postpartum and parenting people to examine birthing people's experiences and attitudes related to infant feeding decision-making among PWH in the US.

## 2 Methods

### 2.1 Design

This pilot study used a modified critical ethnographic approach to examine experiences and attitudes of infant feeding decision-making. Critical ethnography is a methodology originating in critical theory, aimed at understanding how groups of people navigate the social world while also considering how systems of power and privilege repress and inhibit communities (53–55). Due to the exploratory nature of this study, key components of critical ethnography such as participant observation, field engagement, and comprehensive document analysis were not completed. In addition, the theory of intersectionality was used to further guide this research (56–58). Intersectionality is a theory that aims to understand the interconnectedness of social positions, gender, and systems of power and oppression, such as racism, discrimination, and sexism. Human subjects' approval was obtained from the University of California, San Francisco (UCSF) Institutional Review Board (protocol #23-39235). All participants provided written informed consent before participating.

### 2.2 Participants

Participants were recruited through flyers distributed in community and reproductive healthcare settings, social media platforms, and by community organizations serving pregnant and postpartum PWH throughout the US. The inclusion criteria included any pregnant, postpartum, or parenting person currently living with HIV in the US, aged 18–45, and who spoke, read, and could understand English. Due to the nature of a pilot study, the sample size was not preset. Rather, we aimed to include as many participants as possible in the time frame allotted (6 months).

### 2.3 Data collection

Data were collected from October 2023 through March 2024 and consisted of surveys and semi-structured in-depth interviews with participants. Survey questions included demographic information, pregnancy and postpartum support and resources, country of origin, food security, depression, HIV disclosure, and experiences of stigma. The semi-structured qualitative interviews were conducted in English via Zoom by LC and lasted between 60 min and 120 min, with interviews lasting 60 min on average. Participant remuneration consisted of a $50.00 gift card after each interview. Interview questions included infant feeding intentions, pregnancy, postpartum, parenting experiences, healthcare experiences, and participants' involvement in community support groups. The interview guide is detailed in Table 1.

**Table 1:** Semi-structured interview guide.

Category	Interview question
SUPPORT SYSTEM	What kind of support system do you have right now? How does your support make you feel? What kind of community supports do you use (WIC, parenting support group, infant feeding support group)?
INFANT FEEDING MESSAGING	What does infant feeding mean to you? What types of messaging regarding infant feeding have you received?
GUIDELINE CHANGE	What are your thoughts on the changes in the recommendations to provide human milk while living with HIV? How have these changes influenced your thoughts around infant feeding? What kind of discussions have you had with your/your child's providers around infant feeding? Do you feel that you have engaged in shared decision making with your providers? Describe what looked like/hope to look like? What kind of information would be helpful given the new changes?
LIVING WITH HIV	Describe what its like to navigate birth and parenting while living with HIV What advice would you give someone who is pregnant/postpartum/parenting and living with HIV? How has your experiences been with your providers around your pregnancy/care?

### 2.4 Data analysis

Data analysis occurred throughout the data collection process. Analysis began with a grounded theory approach of gerund coding, a method that incorporates line-by-line coding using words in the text to move the concept or word into an action process (59) by LC. Codes were then grouped into themes and concepts. Microsoft Word was used to code transcripts and categorize code excerpts into themes. Themes were then reviewed, and exemplar quotes were populated into a separate Word document for analysis. Themes and concepts were discussed amongst the research team. Memo writing and self-reflexive journaling were used throughout the research process to more deeply engage with the interview content, highlight potential biases, and uncover emerging concepts (60, 61). Open-ended questions within the demographic survey data were analyzed through thematic analysis to identify common themes.

### 2.5 Researcher characteristics and reflexivity

Data analysis was primarily conducted by LC in discussion with SAG and IVA. LC is a white woman, registered OBGYN nurse, certified breastfeeding specialist, and doctoral candidate with experience in qualitative research focused on reproductive health among people living with HIV. SAG is a white woman, a social and behavioral scientist with expertise in qualitative and quantitative research methods focused on the sexual and reproductive health of couples and people living with HIV. IVA is a Black woman, nurse scientist, lactation consultant, and perinatal public health nurse with experience in qualitative and quantitative methods focused on the intersection of racism, systemic and structural barriers, life course perspective, and lactation. The research team engaged in discussions to engage in critical reflection of the interpretation of the themes while considering the positionality of the research team.

## 3 Results

Ten people participated in the study and their demographic information can be found in Table 2. The majority of participants in this study used she/her pronouns and all of them used the term breastfeeding or replacement infant feeding methods (i.e., formula or homemade infant formula). Regarding infant feeding decisions, three participants made their initial decisions before the January 2023 change in infant feeding guidelines. Four participants made infant feeding decisions with their first child before the change, and then made infant feeding decisions with subsequent children after the change in guidelines. Another three participants made infant feeding decisions after the change in the guidelines. Participants in this study provided rich and detailed descriptions of their infant feeding decision-making processes. Infant feeding decisions were not only influenced by infant feeding guidelines, but also by proximity to support, fears of HIV transmission and HIV stigma, and access to feeding options. These influences meet at the intersection of power differences based on gender, social class, and HIV status. From these descriptions, four common themes were identified in the data: the need for support, fear of HIV transmission, the impact of HIV-related stigma, and the desire for infant feeding options, with various subthemes for each.

**Table 2:** Survey demographics (*n* = 10).

Demographics	Sample
Age	Range: 27–40, mean age: 33
Gender	She/He*r* = 9
	She/They = 1
Gravidity	Primipara = 5
	Multiparous = 5
Pregnant	3 pregnant
Postpartum	7 postpartum (1 month to 3 years)
Race/Ethnicity	Haitian American/Caribbean-American = 1
	White = 4
	Black/African American = 3
	Latinx = 2
Education	Some high school = 1
	High school/GED = 2
	Some college/Certificate = 2
	College/AA = 2
	Graduate school or highe*r* = 3
Geographic location	Western US = 8
	Midwest US = 1
	Southern US = 1
	Eastern US = 1
Infant feeding method	Breastfed = 5
	Replacement feeding = 5
Infant feeding before the guideline change	*n* = 3
Infant feeding after the guideline change	*n* = 3
Infant feeding before and after the guideline change	*n* = 4
HIV Infection	Adult = 9
	Perinatal = 1

### 3.1 Support

Support, or lack thereof, was a common theme for all participants, regardless of their chosen infant feeding method. The survey data revealed that most participants reported receiving support through the United States Department of Agriculture (USDA) Special Supplemental Nutrition Program for Women, Infants, and Children (WIC) and the Well Project, a non-profit organization that aims to support women and girls across the gender spectrum living with HIV. Online information sources important to participants included Instagram, YouTube, and Facebook. All participants expressed a need for support, which served as a catalyst for sustaining any infant feeding method.

While feeding intentions were established during pregnancy, all participants expressed a desire for provider support. The intersecting identities of participants' HIV status and expectations of risk management while navigating the medical system during pregnancy left many participants feeling empowered when they received support from their provider. A first-time mother shared the importance of being heard when sharing her intention to provide human milk to her child, who was born the same month of the guideline change.

“…it makes it so much better … and I don't have to hide or lie … as if I'm doing a crime just for feeding my baby. They never made me feel like that, and other women have that experience where they were made to feel like a criminal.”—27-year-old, living with HIV for 3 years, exclusively breastfeeding

Several participants shared experiences of unsupportive providers. Lack of information and support from providers, community-based infant feeding and parenting groups, hospital nursing staff, and lactation support providers generated obstacles to achieving infant feeding goals. A few participants cited a lack of resources, including a shortage of providers in their area or out of their insurance coverage, as barriers to seeking other providers who would be supportive. A participant, who had decided to breastfeed her second child before the change in infant feeding guidelines, described the lack of shared decision-making with her provider and her proximity to providers who would support her decision.

“… one of the providers told me no, that I couldn't breastfeed. I knew that there were options out there, and I wasn't privileged enough to have access to that.”—35-year-old, living with HIV for 8 years, exclusively breastfeeding

Moreover, after the guideline change, participants shared experiences where they were unable to speak up for themselves when discussing infant feeding with their healthcare providers due to fears of discrimination and anticipated stigma. One participant, who made infant feeding decisions after the change in infant feeding guidelines, shared their experience with navigating the healthcare system as a pregnant and postpartum person living with HIV.

“It felt very much like I had to ask for permission, even though it's my body. I feel like because I have HIV, it's not my body, like I have to do what's right, you know, for the world. Meaning, to make myself less than a threat or whatever. A weapon. A criminal.”—27-year-old, living with HIV for 3 years, exclusive breastfeeding

After the guideline change, another participant went to her provider with the desire to breastfeed but was informed it was not an option due to her HIV status. She was unable to change providers due to her geographic location and financial position. Furthermore, she was unaware of the change in guidelines until this study.

“I also wanted to breastfeed, which I couldn't do, and I was trying to figure out if there was a way that I was able to still do that, but they said there's no way that I was able to do that. So he never had breast milk.”—25-year-old, living with HIV for 5 years, exclusive replacement feeding

Most participants expressed the desire for community support from other parents living with HIV. The desire for support from the community was not just to find others with shared identities such as participants' HIV status, gender, and infant feeding modality, but to confirm experiences with others navigating the hospital system, including additional medical procedures and plans of care. One participant who was pregnant with her second baby and decided to breastfeed her first child before the change in guidelines shared how the support from others who are living with HIV provided comfort and empowerment.

“I think hearing like other people doing it [breastfeeding] does make me feel like I'm not crazy. Because, like, clearly they're doing their research and coming to a similar conclusion. It does make me feel good in the sense that it gives me some comfort. And then also … hearing … what were the like repercussions of their decision.”—30-year-old, living with HIV for 7 years, exclusively breastfeeding

Most participants in this study were not aware of any lactation support groups in their area. For those who were aware, participants felt they wouldn't be supported or wouldn't be able to share their experiences due to their HIV status. One participant, who made infant feeding decisions before the change in infant feeding guidelines, sought support from a community lactation support group, resulting in stigma, discrimination, fear, and trauma.

“I disclosed my status, and I was pumping, and flash heating, and feeding my child. I was told by the representative that they just got back from a conference, and during that conference, they were told that if any mother were to disclose a positive status, to call CPS immediately. So, I was like sitting on the bathroom floor, with damaged nipples and a crying daughter. She's hungry. And it was just very, very scary.”—32-year-old, living with HIV for 8 years, exclusive breastfeeding

For participants who chose exclusive replacement feeding, there was a lack of support and information. Many participants who engaged in replacement feeding cited a lack of support from both providers and community organizations. They desired support groups with other parents to learn “tricks and tips” and looked for support in navigating parenthood while living with HIV. One pregnant participant who had decided on replacement feeding after the change in infant feeding guidelines shared that while there was a wide variety of pamphlets regarding breastfeeding, there was very little information on safe replacement feeding practices and peer support groups, leaving them feeling left out.

“Seeing all those papers for like breastfeeding and you know, people are like oh, you're gonna breastfeed or like oh, them just kind of assuming your breastfeeding. Which is fine like I understand. But you know sometimes it's just like a hard reminder of like what you can't do. Or like, what you could do but, you know, there's some risk to it. So … it could make me feel, you know, like a little sad. But then I reassure myself that you know, like that's not something I wanted to do.”—28-year-old, living with HIV for 28 years, exclusive replacement feeding

### 3.2 Fear of HIV transmission

For all participants, fear of transmission was a major factor in infant feeding decisions. For those who decided to exclusively replacement feed, fear of HIV transmission was the driving factor. Moreover, maternal identity shaped infant decisions such that participants saw it as their maternal responsibility to protect their infant from harm. One participant, who made infant feeding decisions before and after the infant feeding guidelines change, shared that exclusive replacement feeding was the safest way to eliminate risk of transmission.

“I really wanted to breastfeed both of them, you know, but I was told that it, there's a possibility of transfer. And I was really kind of sad about that. I would never, you know, do that to my kids.”—38-year-old, living with HIV for 8 years, exclusive replacement feeding

For one participant who was breastfeeding before the change in infant feeding guidelines, fear of transmission contributed to anxiety and guilt for placing their child at risk that continued well beyond the cessation of breastfeeding.

“…there still was that anxiety and a little bit of guilt, too, because I felt like it was my fault. Even though I knew I was doing this because my milk was the best thing for my baby. And even after the cessation of breastfeeding with both of my children, if they got a cold, I was like oh my god, do we need to get her tested again?”—32-year-old, living with HIV for 8 years, exclusive breastfeeding

Fear of transmission was present for all participants. One participant, who was pregnant with her second baby and breastfed her first child before the change in infant feeding guidelines shared that despite her confidence in her undetectable viral load, the anxiety of breastfeeding remained.

“Yeah, so I had an anxiety about obviously transmitting the virus to my children, even though I knew the signs. I knew I was undetectable.”—40-year-old, living with HIV for 8 years, exclusive breastfeeding

### 3.3 HIV stigma

Participants in this study described experiences of anticipated, experienced, and internalized HIV stigma while making infant feeding decisions. Anticipated and experienced stigma from providers and the community shaped how participants accessed support and was a significant factor in their infant feeding decisions. Internalized HIV stigma included fear of disclosing their HIV status, and the desire to feel like they were making a normative infant feeding choice.

#### 3.3.1 Anticipated stigma

Most participants anticipated stigma from providers which impacted how they were going to feed their baby. Before the change in guidelines, one participant described the anticipated stigma they felt when discussing their infant feeding decisions with their provider. This anticipated stigma influenced their decision to replacement feed. However, shortly after giving birth, they exclusively breastfed.

“So my first challenges were that I had not made the decision that I was going to breastfeed until the very end actually after I gave birth. My plan was to formula feed, and it was due to stigma. Due to fear bringing up the conversation.”—32-year-old, living with HIV for 8 years, exclusive breastfeeding

#### 3.3.2 Internalized stigma

For participants who chose exclusive replacement feeding, sharing their decision with family members and friends could result in unwanted disclosure. For participants who had a family culture of breastfeeding, the decision to replacement feed met at the intersection of their HIV status and their family values. Many participants who decided to replacement feed described a tactic for publicly negotiating replacement feeding in a “breast is best” setting. This often led to generating a script to explain their decision to replacement feed that would be socially acceptable. One participant who made infant feeding decisions before and after the infant feeding guideline change shared what she told people when they discovered she was replacement feeding.

“Mostly everybody has always breastfed. And that's just the way everybody has always done it. And I have this friend, actually. She asked me, are you going to breastfeed? And she doesn't know my status, you know. And I said, no … I'm gonna bottle feed. And she's like, why? She was so against it. You need to breastfeed, so it's healthier. And I was like … well, no, cause they grow teeth early *giggles* like they grow too early. I don't want them to break my nipples *giggles*.”—38-year-old, living with HIV for 8 years, exclusive replacement feeding

Many participants in this study described feelings of internalized stigma. For those who were breastfeeding, these feelings influenced infant feeding experiences including feelings of guilt and anxiety. For one participant who breastfed both of their children before the infant feeding guideline change, this anxiety shaped her breastfeeding experience.

“But it's an ongoing process to be able to like really believe that. And then when my children were involved, it very much sometimes felt like, well, if I was negative, it wouldn't matter. I wouldn't have this anxiety.”—32-year-old, living with HIV for 8 years, exclusive breastfeeding

Several participants shared the desire to be seen as normal. Many participants expressed the desire to been seen women and parents. Finding their voice as a parent living with HIV was experienced by a first-time mom making infant feeding decisions after the change in infant feeding guidelines.

“So there's not much of a difference. I'm still a woman, I still produce the same amount (of milk) as someone without HIV and the quality of the breastmilk is still magical and like powerful stuff”—27-year-old, living with HIV for 3 years, exclusive breastfeeding

For some participants, the lack of infant feeding options left them feeling their identity as parents was invisible against their HIV status. One pregnant participant shared their experience of not having infant feeding options with her two older children.

“So with my first 2 pregnancies, I was told that you know there's no there's no chance for you to breastfeed so I was like, with my first, I was like a little sad where I was like, man, I wish I could breastfeed, you know. To have that, that experience or you know like I felt like maybe I wasn't also like a good mom. Like a normal mom where I could breastfeed.—28-year-old, living with HIV for 28 years.

However, some participants felt that their infant feeding method allowed them to step away from their HIV status and identify as a mother. For many participants, breastfeeding was centered around motherhood. One participant, who made infant feeding decisions before the change in guidelines, shared their feelings of normalcy and activism while they were breastfeeding.

“I guess there is a part of me that felt like because I was having this breastfeeding relationship, I didn't have to like directly identify with my positive status. I was doing the same thing like any other mother could do without a barrier. You know, aside from the fact that they were on prophylaxis. And so there was something about like being in a public place and like being able to like openly breastfeed because there's activism around that too.”—32-year-old, living with HIV for 8 years, exclusive breastfeeding

#### 3.3.3 Experienced stigma

Most participants in this study described experienced stigma from various sources. These experiences influenced how they reached out for support and with whom. For one participant who breastfed both of her children before the change in infant feeding guidelines and was supported by her provider, she experienced stigma from a family member who shared their status with another healthcare professional in the attempt to stop them from breastfeeding.

“[They called me to] try to talk to me out of breastfeeding, while I was breastfeeding you know. So I've had, yeah, all sorts of stigma and just, you know, negative feelings, people disconnecting from me. It doesn't feel good. It doesn't feel good and those are times where I wish that it wasn't a factor in my life, but it is, you know. It is.”—32-year-old, living with HIV for 8 years, exclusive breastfeeding.

For participants who were exclusively breastfeeding, finding support was a significant challenge. One participant who decided to breastfeed her first child with the support of her providers before the change in infant feeding guidelines, described the stigma she experienced while seeking support from an in-patient lactation consultant after delivery.

“The lactation consultant refused to see me because I had HIV. And she herself didn't believe that I should be breastfeeding. She was literally refusing me services.”—30-year-old, living with HIV for 7 years, exclusively breastfeeding

### 3.4 Infant feeding options

All participants expressed a desire to discuss infant feeding options with their providers. Fewer than half of the participants in this study were aware of the change in infant feeding guidelines. For those participants who felt they engaged in shared decision making with their providers and felt secure in their infant feeding decisions, they still desired to have conversations with providers about the risk of transmission, and the benefits of both replacement feeding and breastfeeding. For one participant who made infant feeding decisions before and after the change in guidelines, having the opportunity to discuss infant feeding options despite their infant feeding intention was important.

“I think I just would have liked more information on it altogether, even though I chose bottle feeding. I think it would have been nice to have … all the information.”—38-year-old, living with HIV for 8 years, exclusive replacement feeding

Furthermore, all participants expressed happiness with the infant guideline changes, despite how they chose to feed their babies.

“So I feel like it's good that it's progressing that way that people could breastfeed now. And Yeah, like, yeah, like I'm glad that that's possible.”—29-year-old, living with HIV for 6 years, exclusive replacement feeding

For some participants, having the option to breastfeed was a journey filled with healing and joy.

“It's an opportunity to feel like great about your body and what it can do and like you're not toxic to your child, you know. I can make this liquid gold … regardless of my status. And that's so empowering and beautiful … it's healing  …  It heals you. It was healing for me. Because I was able to meet the needs of my child, regardless of my status.”—32-year-old, living with HIV for 8 years

## 4 Discussion

The results of this pilot study identify the infant feeding experiences of perinatal and parenting PWH in the US. Additionally, viewing these experiences through an intersectional lens allows for the consideration of missed opportunities in support and care for birthing PWH in the US. All participants in this study sought support from various sources regarding their infant feeding decisions. However, financial positionality and geographical location had the largest impact on access to supportive providers. Fear of HIV transmission was also a significant factor in infant feeding decision-making, and for some, often intersected with beliefs about the benefits of breastfeeding and the role of mothers to protect their infant from harm. Furthermore, all participants had experienced and anticipated stigma regarding their infant feeding decisions, and this shaped how they engaged with providers and community support systems. Despite how people decided to feed their infant, all participants wanted to feel that their decision was normal. Finally, while infant feeding decisions were made in pregnancy, all participants wanted information on various infant feeding options. Current literature from the US on infant feeding decisions report similar findings, including fear of HIV transmission and the benefits of support towards any infant feeding modality (49, 50). The findings from this study contribute to the limited data on infant feeding among PWH in the US and can help inform targeted supports and professional educational opportunities for healthcare and lactation support providers to make positive changes to how women and birthing PWH are supported in their infant feeding decisions.

The most significant facilitator to infant feeding decisions was support from providers, family, and other parents living with HIV. There is robust evidence that suggests support plays a critical role in the initiation and duration of breastfeeding for all women (62–65). For PWH, support from family members and partners has been shown to facilitate breastfeeding (49). Moreover lactation consultants have demonstrated a positive impact on lactation initiation and lactation rates in the US and may be a facilitator to breastfeeding among PWH in the US (66). However, there were participants who decided to replacement feed that felt left out of infant feeding conversations and lacked information. Recent evidence from the global community highlights a lack of support for parents who are replacement feeding, including education on safe replacement feeding practices (67–69). Moreover, participants in this study expressed the need for support from others who identify and understand the experience of making infant feeding decisions while living with HIV. This suggests that tailored and community-based models of support can facilitate infant feeding decisions as well as address the complex nature of parenting while living with HIV.

Fear of transmission was a significant factor when considering any infant feeding method for all participants. However, fear of transmission intersected with the view that the role of a mother is to protect their child from harm, which resulted in feelings of sadness and guilt. A study conducted in India found that mothers who feared HIV transmission experienced internalized stigma rooted in the moral judgment surrounding their roles as mothers (70). Moreover, feelings of guilt and sadness due to fears of transmission were also discussed in studies from the global community (71–73). For participants who replacement fed, fear of transmission was the primary reason not to breastfeed. For those who chose to provide human milk, fear of transmission was a motivator to exclusive breastfeeding despite ongoing stress. Fear of HIV transmission within the context of human milk feeding has been well documented within the global community (74–80). Providing evidence-based information about the risks of HIV transmission of breastfeeding with an undetectable viral load through different care systems may address fears of transmission (49, 73).

Participants in this study experienced various forms of HIV stigma, including anticipated, internalized, and experienced stigma from providers, family, and the broader community. All participants anticipated stigma from either providers, community members or family. This stigma impacted participants' infant feeding options and decisions around HIV status disclosure. There is evidence that suggests HIV stigma creates a barrier to engaging in a shared decision-making model with providers, including discomfort about discussing treatment plans, power imbalances that favor providers, and a desire not to be seen as a “difficult” patient (81–83). Participants who were breastfeeding described experiences of stigma including criminalization, which furthered feelings of internalized stigma surrounding human milk feeding. This highlights the impacts of historical discrimination and trauma related to infant feeding and reproductive choices on present infant feeding decision-making for PWH (26). Recent evidence suggests a trauma informed care approach by providers may decrease the impact of trauma experienced by PWH throughout the antenatal and postpartum time period (84).

Many participants expressed the importance of feeling like their infant feeding decisions were normal which facilitated stigma-free decision-making regarding infant feeding. This allowed participants to step away from their HIV diagnosis, and more specifically, feelings of discrimination and stigma, to focus on how they wanted to feed their babies. There is evidence of increased coping skills while living with HIV and pregnant and parenting, including finding refuge in caring for babies, and continued engagement in HIV care, which can combat internalized stigma (85–87). For participants in this study, the ability to breastfeed, especially in public spaces, enabled them to embrace their identity as parents, free from the stigma of a virus that was once considered a barrier to breastfeeding. Some participants who decided to replacement feed expressed feelings of sadness in their decision, often citing the desire to be a good mother. Several studies have found that PWH have struggled with the intersection of motherhood and infant feeding while not breastfeeding (49, 69, 78, 80, 88–90). While most of the participants in this study were happy with their infant feeding decisions, all of them faced challenges with the intersection of motherhood and their HIV status.

Participants in this study desired information from providers on all infant feeding options despite having made prior decisions on infant feeding methods. However, participants reported that some providers withheld support and infant feeding information. Recent studies have found concerns among providers regarding infant feeding options. Providers have cited obligations to eliminate the risk of HIV transmission, lack of substantial data, and ambiguity in infant feeding guidelines as challenges, resulting in some providers not recommending breastfeeding to PWH. This has led to a lack of informed choice among PWH and also highlights barriers to shared decision-making with providers (91, 92). The current literature highlights gaps in the implementation of the shared decision model when making infant feeding decisions, including a lack of interdisciplinary support among providers (49, 50, 71, 93). In a survey conducted by the Well Project, providers supported the change in infant feeding guidance, but cited institutional barriers, including the lack of explicit policies that support breastfeeding among PWH (93). These findings reveal gaps in shared decision-making while emphasizing the need for the adoption of evidence-based infant feeding policies among institutions that prioritize experiences of PWH, including healthcare provider training.

## 5 Limitations

This study had some limitations. Although this was a national study, most participants resided in western US states. Therefore, findings may be more representative of the experiences of PWH from the Western states. Future research should seek to have a broad and even geographic distribution to better compare the experiences of PWH across the US. Furthermore, while this study utilized an intersectional lens, questions exploring the role of race and ethnicity on infant feeding decisions were not asked. Therefore, the research team was unable to confirm racial identities as an influence on infant feeding decision-making. Additionally, despite the study's modest sample size, we identified recurring themes in the data. However, we may not have reached data saturation due to the pilot nature of this study. While we know that all women who took part in the study were on ART, we do not know if they were virologically suppressed. Therefore, we cannot speak to whether the information given by providers about infant feeding options or HIV risk was correct or incorrect. Regardless of whether the participant was viremic or not and whether the information given by the provider was medically accurate, some participants described feeling unsupported by their providers. Finally, participation bias may also be present, as this was a sample of people willing to share their infant feeding decisions, who had time to participate in an interview, and had reliable internet access.

## 6 Conclusion

Infant feeding decisions for those living with HIV are complex and are influenced by personal experiences, families, communities, and systems. Given the significance of human milk feeding for the maternal-infant dyad, supporting individuals living with HIV to meet their infant feeding goals should be a top priority. Implementing supportive policies and practices requires centering the needs of PWH. While this study further contributes to the current research on infant feeding decisions among PWH in the US, more research on the needs of those making infant feeding decisions while living with HIV in the US is needed. The US has an opportunity to support lactation for PWH and to change course with an equitable reproductive justice lens through a culturally appropriate, trauma-informed care approach fueled by evidence and not fear.

## Data Availability

The original contributions presented in the study are included in the article/Supplementary Material, further inquiries can be directed to the corresponding author.
